# MicroRNA Profiling in Circulating Exosomes in Oral Squamous Cell Carcinoma: A Systematic Review

**DOI:** 10.7759/cureus.43235

**Published:** 2023-08-09

**Authors:** Dinesh Y, Pratibha Ramani, Monal Yuwanati, Karthikeyan Ramalingam, Gheena S

**Affiliations:** 1 Oral Pathology and Microbiology, Saveetha Dental College and Hospitals, Saveetha Institute of Medical and Technical Sciences, Saveetha University, Chennai, IND

**Keywords:** oral potentially malignant disorders, microrna, systematic review, oral squamous cell carcinoma, circulating exosomes

## Abstract

Oral squamous cell carcinoma (OSCC) is the most common head and neck cancer with several risk factors. Exosomes are extracellular vesicles generated by the fusion of multivesicular structures with the cell membrane and play an important role as intercellular messengers. MicroRNA (miRNA) is a noncoding RNA and regulates post-transcriptional modification. The present systematic review aims to identify and correlate the possible association and role of circulating exosomes with OSCC. Using the search strategy, articles fulfilling the inclusion criteria, published between January 2012 to March 2022, were retrieved from online databases including PubMed, Scopus, Web of Science, and Cochrane Library. About 904 articles were found using an electronic database and a human search. After reviewing the titles and abstracts, 614 studies were eliminated, and duplicate articles were removed. Five studies were included in this systematic review. Circulating exosomal expression of miRNA27, miRNA 21, and miRNA 155 showed significant upregulation in OSCC patients. Circulating exosomes could be potential biomarkers to be used in the detection of patients with OSCC. More studies are warranted in this area to gain a better understanding of the pathophysiology of OSCC and the function of molecular markers from circulating exosomes. Understanding the role of molecular markers from circulating exosomes in pathogenesis will provide a better understanding of the development of the disease, necessitating more study in this area. According to this review, circulating exosomes might be a potential approach to the identification of OSCC.

## Introduction and background

More than 90% of head and neck cancer is contributed by oral squamous cell carcinoma (OSCC) [1]. Several risk factors for OSCC have been established, including smoking, alcohol intake, use of smokeless tobacco, and infection with human papillomavirus [1]. Despite advances in cancer care, oral cancer patients have a five-year mortality rate of about 50% in different stages of the disease [2,3]. Advances in early diagnosis and more personalized therapy to minimize morbidity are being investigated globally on oral cancer patients globally [4].

Exosomes are extracellular vesicles ranging from 30 to 150 nanometers in size, formed from the fusion of multivesicular bodies with the cell membrane [5,6]. They play a role both in physiological and pathological conditions [7,8]. These vesicles can be found in almost all human body fluids like plasma, serum, saliva, cerebrospinal fluid, and urine [9]. Exosomes consist of protein cargo and genetic material like DNA and microRNA (miRNA) [10]. These molecules can be transported to recipient cells, act as intercellular messengers, and regulate their signaling pathways [11]. Large amounts of exosomes are secreted by tumor cells, which aid in tumor progression [12]. Exosomes are a key element of the tumor microenvironment and one of the most important factors in the development and metastasis of tumors, contributing a wide range of molecules that could be implicated in cancer pathogenesis as well as transferring genes to the germline [13]. Tumor-derived exosomes regulate cellular metabolism by angiogenesis, immune suppression, metastasis, cancer-associated fibroblast activation, and extracellular remodeling [14,15]. The term “exosomes” used in research publications refers to isolated extracellular vesicles; it is now well known that most traditional and innovative purification methods showed stable and reliable results [16-18]. Over the past decade, numerous research indicates that exosomes are playing an important role in the diagnosis, prognosis, and treatment of head and neck cancer [19,20].

Several biomarkers have been studied to aid in diagnostic purposes, of which miRNAs are the most commonly studied biomarker. MiRNAs are made of 18-25 nucleotides of noncoding RNAs and, through complementary binding to the 3′ untranslated regions (3′UTR) of target mRNA, regulate post-transcriptional gene expression [21]. MiRNAs influence signaling pathways by targeting mRNA [22]. MiRNAs have been shown to regulate several cellular activities, constituting a complex regulatory network [23,24]. Utilizing liquid biopsy miRNAs as a diagnostic tool in biomarker programs is the future [25]; practicality and chairside application must all be taken into account and maximized [26]. Identifying particular miRNAs, on the other hand, may shed light on the process of oral cancer illness and pathophysiological alterations and uncover possible treatment targets [16,17].

This systematic review is aimed to explore the role of circulating exosomes as a reliable biomarker in OSCC.

## Review

Materials and methods

The Preferred Reporting Items for Systematic Reviews and Meta-Analyses (PRISMA) guidelines 2020 for systematic review were followed in this systematic review [27]. The PROSPERO registration number for this systematic review is CRD42021248804.

Search Strategy

To collect relevant publications published in the last 10 years, the authors undertook a literature search in PubMed, Scopus, Web of Science, and the Cochrane Library. A manual search was done by the authors to retrieve the additional studies assessing the association of circulating exosomes in OSCC up to March 1, 2022. The following keywords were used for the search of articles: (extracellular vesicles or exosomes or microvesicles or ectosomes or shedding vesicles or microparticles or oncosomes or cell-derived microparticles or nanovesicles) AND (pharynx or oropharynx or nasopharynx or throat or oral or mouth or palate or tongue or floor of mouth or lingual mucosa or buccal mucosa or lip or labial mucosa or tonsil or mucosa or retromolar or cheek or gingiva or vermillion border) AND (premalignant or potentially malignant or pre-cancer or neoplasm or cancer or malignant or tumor or carcinoma). Filters focused on the search from Jan 2012 to March 2022.

Eligibility of studies

The title and abstract of retrieved articles were evaluated to assess their eligibility as per inclusion and exclusion criteria.

Inclusion Criteria

The inclusion criteria include clinical patient studies diagnosed with OSCC, studies that evaluated the circulating exosomes (in body tissues/serum/plasma) prior to any intervention such as surgery, drug treatment/chemotherapy, or radiotherapy, studies that provided circulating exosome expression profiles and clinicopathological and demographic data, and only English-language articles published in the last 10 years.

Exclusion Criteria

In-vitro and animal studies, literature and systematic reviews, meeting abstracts, animal studies, pilot studies, case reports, and case series were excluded.

Screening and Selection

Two reviewers, PR and MY, screened the titles and abstracts of articles for eligibility. After removing ineligible articles, full texts were retrieved and checked for inclusion. Cross-references to these articles were searched for additional studies.

Data Extraction

Two reviewers, PR and MY, finalized the articles and performed data extraction. The following data were extracted: year of study, age, gender, author, samples, specimen, type and level of expression of exosomes, and method detection or estimation. Data were individually extracted from all qualifying studies. The data were extracted into a preset Microsoft Excel (Microsoft, Washington, USA) file for subsequent research quality evaluation and data synthesis.

Data Analysis

For the purpose of summary synthesis, the studies were grouped based on OSCC. There were variations in specimen type used for the estimation of exosomes. Hence, descriptive analysis was performed. The findings were presented as mean (SD/SE/95% confidence interval), frequency, and percentage.

Risk of Bias and Quality Assessment

Two reviewers, PR and MY, independently and in duplicate examined all of the included papers for study design characteristics and internal validity aspects. The risk of bias was assessed by assigning a score of low, high, or uncertain to each included study. Each study’s overall quality was then appraised by evaluating the six bias categories. A score of 3, 1, or 0 indicated a low, uncertain, or high risk of bias, respectively. Nonrandomized studies tool Rob 1 was used to assess the risk of bias. To assess the quality of the results supporting documentation, GRADEpro (Evidence Prime, Hamilton, Ontario) was employed [28]. The likelihood of bias, indirectness, inconsistency, imprecision, and other characteristics all play a role in establishing the quality of the evidence (+, very low; ++, low; +++, moderate; ++++, high).

Results

Search Details and Study Selection

**Figure 1:**
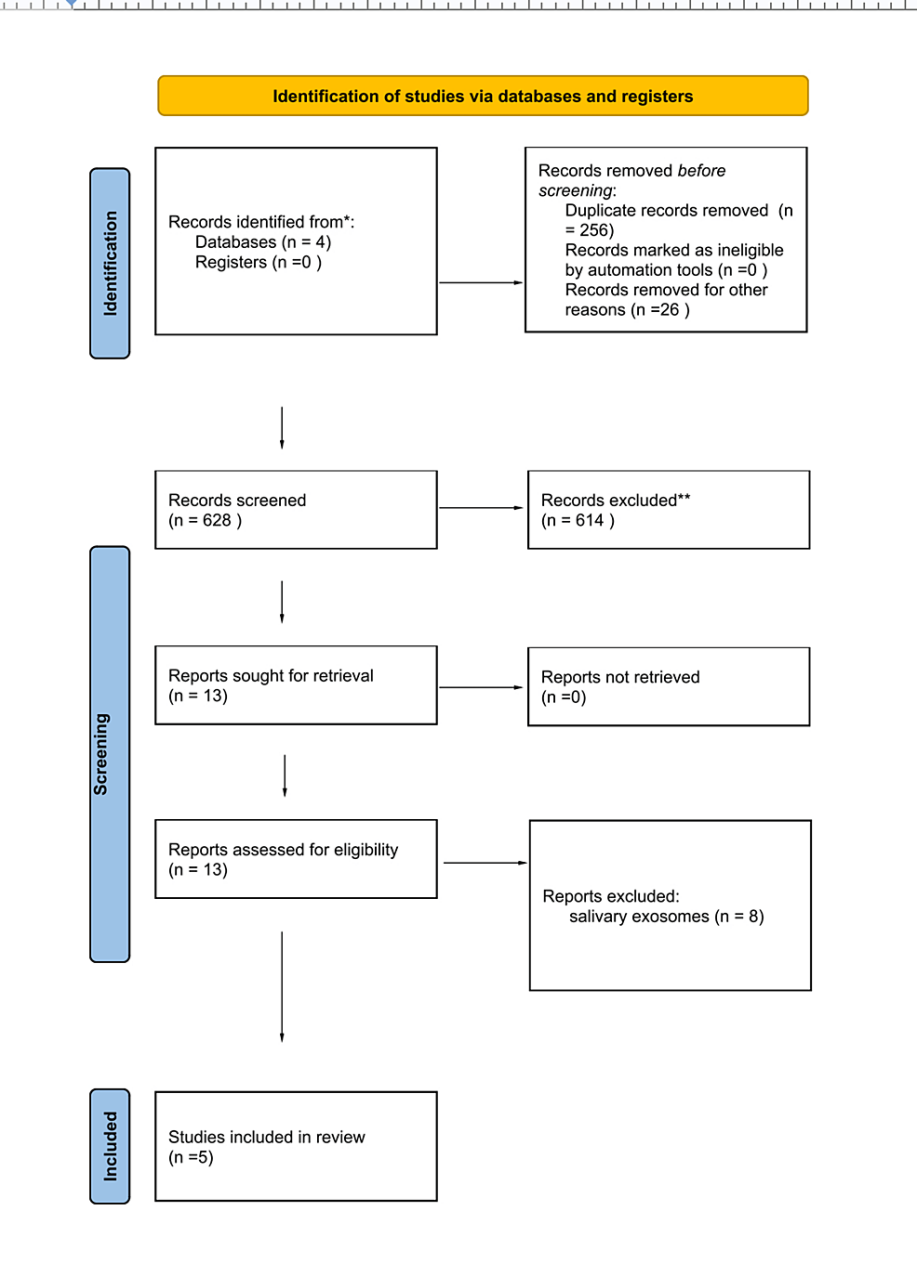
PRISMA 2020 flow diagram showing Included articles

The search yielded 910 articles from databases; 628 articles were selected for screening after removing duplicates (n = 256) and non-relevant articles (n = 26). After title and abstract screening, 614 were further excluded, and only 13 articles were included for full-text screening. Finally, five articles were found eligible for this review. These articles were published between 2016 and 2020. The eligibility of each article was determined based on the title, abstract, and full-text reading. The study selection and screening process are provided in the PRISMA flow diagram (Figure 1).

Characteristics of Included Studies

The present systematic review was reported according to PRISMA guidelines 2020 for systematic review. The data from all studies included in the systematic review are summarized. The characteristic data of included studies were presented in Table 1. A case-control study, cross-sectional study design of pooled sample size OSCC (n = 159); control/healthy (n = 50). Forty of the studies were done on the Chinese population, 40% of the studies were done in North America, and 20% of the studies were done on the Spanish population. Sixty percent of the studies used plasma, and the remaining 40% used serum as samples for the estimation of exosome levels and types. As most included studies did not provide the values for the level of expression of circulating exosomes, a meta-analysis was not performed.

**Table 1:** Characteristic data of identified five studies that describe molecular markers in the diagnosis of OSCC OSCC: oral squamous cell carcinoma, miRNA: microRNA, NM: not mentioned, NA: not available

Study	Population	Study period	Sample number (patient/control)	Source of miRNA	miRNA/dysregulation	Cancer site	Histological stage	Tumor/lesion grade	Lymph node metastasis	miRNA analysis platform	Follow-up
Luo et al. 2020 [29]	China	July 2014 to July 2019 (5 months)	108 OSCC and 50 healthy	Serum	cric_0000199	NA	Well: 8 high, 7 low; moderate: 33 high, 21 low; poor: 27 high, 12 low	TNM I or II: 33 high, 28 low; III or IV 35 high, 12 low	N1-3: 39 high cric_0000199; 14 low cric_0000199	qPCR	1,3,5 years follow up
Jiang et al. 2019 [30]	China	NM	3 OSCC	Serum	28 proteins esp. PF4V1, XCXL7, F13A1, ApoA1	NA	NA	NA	NA	Western blot, RTqPCR, IHC	
Zorrilla et al. 2019 [31]	Spain	6 October 2014 to 23 April 2015 (7 months)	10 OSCC	Plasma	CD63; CAV-1	Mouth floor: 3, upper jaw: 3, palate: 1, jugal mucosa: 1, tongue base: 1, RMT1	Moderately: 8, poorly: 2	T4M0	N0: 5, N1: 2, N2: 1, N3: 2	Immunocapture-based assay, western blot	October 2014 to March 2018
Heravi et al. 2018 [32]	USA	NM	34 OSCC	Plasma	miR21, miR27b, miR27a	NA	NA	NA	NA	TaqMan miRNA assays	NA
Rabinowits et al. 2017 [33]	USA	NM	5 OSCC	Plasma	16 miRNA, 9 up, 7 down	Tongue	NA	Stage II-IVa	NA	TaqMan-based miRNA profiling	NA

Summary of Findings

**Figure 2:**
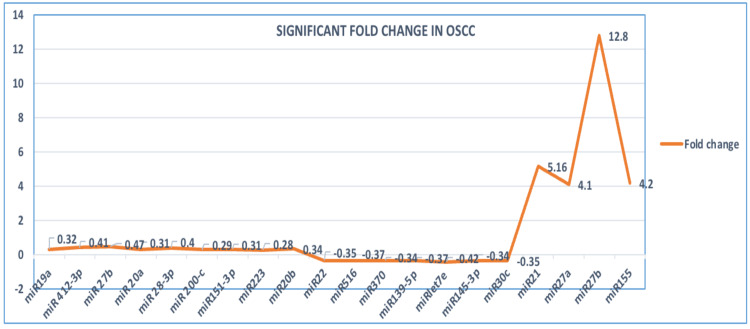
Line diagram showing significant fold change in upregulation and downregulation expression of miRNA in OSCC patients OSCC: oral squamous cell carcinoma

The summary of the included studies is presented in Table 2. A total of 160 OSCC cases and 50 healthy individuals as controls were included in the study. Two studies were in the United States, two studies were in China, and one study was in Spain. Of the 359 miRNA analyzed, 23 miRNA were differentially expressed. Forty percent of the studies used ultracentrifugation for exosome isolation, 40% of studies used an exoEasy Maxi kit (Qiagen, Hilden, Germany), and 20% of studies used an ExoQuick serum exosome isolation kit (System Biosciences, Palo Alto, California, USA). Multiple methods were used for the characterization of exosomes: 60% of the studies used transmission electron microscopy, and 40% of the studies used CD63 markers. Forty percent of studies used scanning electron microscopy and nano-tracking analysis. For molecular analysis used in these articles, one study was done using microarray, three studies were done on quantitative polymerase chain reaction, and one study was done on immunocapture. All of the studies (100%) found a significant difference in the expression of the molecular markers studied in patients with OSCC versus healthy controls. OSCC (20%) upregulation of miRNA 21, miRNA 155, and miRNA27a with a fold change of 5.16, 4.2, and 4.12 respectively. OSCC (40%) upregulation of miRNA27b with a fold change of 12.8 and −0.41 (Figure 2). Significant downregulation of miRNA was also found in 40% of studies in OSCC. The circ_0000199 levels in circulating exosomes in patients with OSCC were significantly increased (P < 0.001) when compared to healthy individuals. Patients with OSCC who had lymph node metastases had 37 proteins that differed from those of healthy controls. All the studies’ outcomes were assessed statistically with a confidence interval of p < 0.05. In the majority of the studies, only the upregulation or downregulation of the molecular markers was assessed.

**Table 2:** Summary of the included study miRNA: microRNA, OSCC: oral squamous cell carcinoma, SCC: squamous cell carcinoma, qPCR: quantitative polymerase chain reaction

S. No	Author and year	Sample size	Specimen type	Markers	Analysis
1	Luo et al. 2020, China [29]	OSCC (n=108), healthy (n=50)	Serum	circ_0000199	qPCR
2	Jiang et al. 2019, China [30]	OSCC (n=3)	Serum	Protein	Laser chromatography-mass spectrometry, qPCR
3	Zorrilla et al. 2019, Spain [31]	OSCC (n=10)	Plasma	CD 63, Cav-1	Immunocapture
4	Heravi et al. 2018, USA [32]	SCC (n=34)	Plasma	Quantification, miRNA	Nanoparticle tracking analysis, qPCR
5	Rabinowits et al. 2017, USA [33]	Tongue - SCC (n=5)	Plasma	miRNA	Microarray

Summary of Risk of Bias Assessment and Quality Assessment of Included Studies

**Figure 3:**
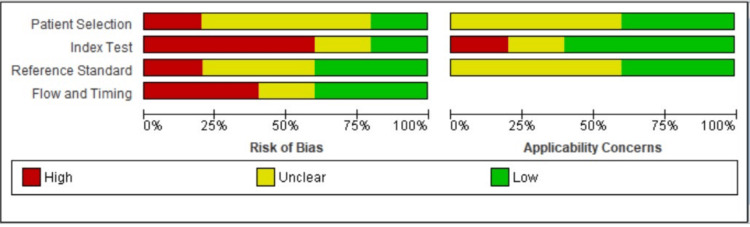
Risk of bias of included study

**Figure 4:**
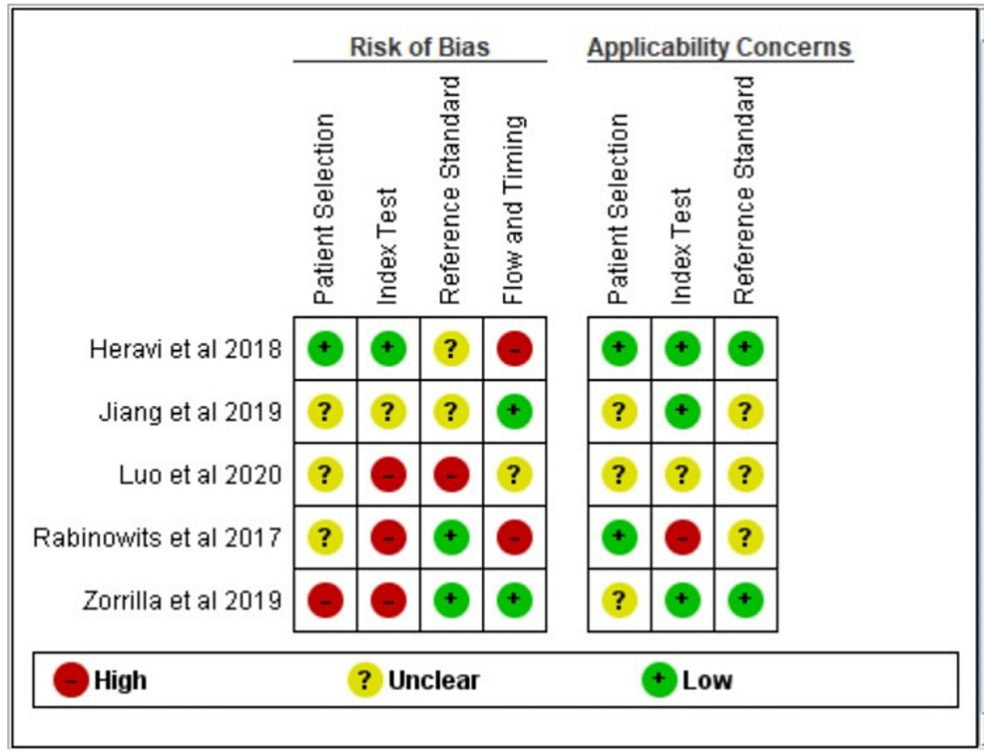
Risk of bias assessment

A non-randomized study tool Risk of Bias 1 (Cochrane, London, England) was used to assess the risk of bias. Reference standard and index test was the most common risk of bias among the studies (Figure 3). Most of the studies showed a moderate to high risk of bias, and one study showed a low risk of bias (Figure 4).

On assessing the risk of bias using ReVMan 5.4.1 (Cochrane, London, England) for the included studies, four studies showed high risk, and one study showed unclear risk. When assessing the quality of evidence by GRADEpro software, due to the heterogeneous nature of studies, it was not possible to combine effects and derive any conclusion (Table 3).

**Table 3:** The GRADEpro approach The level of evidence according to GRADEpro (grading of recommendations, assessment, development, and evaluations) approach. Overall quality of evidence (+, very low; ++, low; +++, moderate; ++++, high) [28]

Certainty assignment							Summary of findings					Importance
No. of studies	Study design	Risk of bias	Inconsistency	Indirectness	Imprecision	Other considerations	No. of patients		Effect		Certainty	
							[Intervention]	[Comparison]	Relative (95% CI)	Absolute (95% CI)		
New outcome												
5	Observational studies	Serious	Not serious	Not serious	Serious	Strong association, all plausible residual confounding would reduce the demonstrated effect of a dose-response gradient	OSCC (33.33%) upregulation of miR27B with a fold change of 12.8 and -0.41. Significant downregulation of miRNA was also found in 33.33% of studies in OPMD/OSCC. About 16.66% of studies showed downregulation of miR301b-3p and miR144-3p with fold changes of 0.35 and -0.47.circ_0000199 levels in circulating exosomes in patients with OSCC were significantly increased (P < 0.001) when compared to healthy individuals. 0SCC patients with lymph node metastasis had 37 different proteins than stable controls.				⨁⨁⨁◯ moderate	Important

Discussion 

Cancer patients, including those with oral cancer, have been documented to have circulating exosomes in biological fluids such as serum and saliva. Exosomes contain a large number of miRNAs, which have a regulatory role in cancer progression by controlling cellular processes such as cell migration, apoptosis, angiogenesis, and so on. Because dysregulation in the expression of miRNA can be detected in biological fluids even before the clinical manifestations, identification of these can be advantageous in early diagnosis [26].

The systematic review found that there was an upregulation of nucleic acid and protein expression in circulating exosomes with OSCC. These include miRNA, circular RNA, proteins, and so forth. OSCC represents 90% of oral malignancies, and most of these are due to the malignant transformation of oral potentially malignant disorders (OPMDs) [29]. There are various identified circulating exosomes in saliva and plasma in OSCC (Table 2). Five articles analyzed the expression of molecular markers derived from circulating exosomes in 160 OSCC patients compared with 50 control patients. All five studies supported circulating exosome association with OSCC. Although no single exosome is strongly associated with OSCC, few are expressed more strongly in OSCC than in normal individuals. Heravi et al. studied miRNA expression on 34 OSCC patients using qPCR. Increased miRNA21, miRNA27b, and miRNA27a increased expression in extracellular vesicle fraction versus nonfraction in OSCC patients. In OSCC patients, salivary extracellular vesicles miRNA24-3p, miRNA 512-3p, and miRNA-412-3p were unregulated compared to control patients. MiRNA 24-3p promoted cancer cell growth and invasion, and it inhibited apoptosis by targeting menin and SRY-related HMG-box genes (SOX 7) gene miRNA 512-3p inhibit the Mucin 1 (MUC1) gene [30]. MiRNA 512- 3p inhibits MUC1 which is responsible for tumor proliferation and invasion [31]. The circ_0000199 levels in circulating exosomes were considerably higher in OSCC patients when compared to healthy individuals in 20% of studies [31] and were substantially greater in OSCC patients with advanced stage than with early stages; the expression was associated with habits, size of lesion, and nodal metastasis. The circ_0000199 upregulation in circulating exosomes from OSCC patients is related to poor survival results. There was increased extracellular vesicle number and size in OSCC patients when compared to control patients [33]. When lipopolysaccharides and ethanol were added in varying dosages to the oral adenosquamous carcinoma cell line (CAL-27) OSCC cell culture, the findings revealed an increase in extracellular vesicle production, though the miRNA cargo of extracellular vesicles did not appear to change significantly in 16.66% of the studies [34].

During carcinogenesis, tumor hypoxia develops, forcing the tumor cell to initiate glycolysis to meet energy requirements for growth through the hypoxia-inducible factor. It alters the glucose influx in the cell and lactate production through glucose transporter 1 (GLUT1), pyruvate dehydrogenase kinase 1 (PDK1), and monocarboxylate transporters (MCT4). This results in higher expression of GLUT1, PDK1, and MCT4 and lower expression of Caveolin 1, indicating the tumor and stromal cell interplay in tumor growth. PDK1 expression regulates the HOXA11 gene and inhibits miRNA518a-3p to promote tumor proliferation and invasion in OSCC. It promotes cell migration and cell regulation in phosphatase and tensin homolog (PTEN) gene deficiency. Expression of miRNA in extracellular vesicles was derived from tongue OSCC from five patients and plasma samples by Rabinowits et al. [34]. miRNA27b regulates mesenchymal-epithelial transition factor (MET) oncogene in OSCC, enhancing the proliferation and aggressiveness of the tumor, which was found to be overexpressed. The expression of miRNA27b may suggest aggressiveness and recurrence of OSCC [34]. Human gingival fibroblasts (HGFs) had tumor microvesicles (TMVs) incubated with OSCC-derived microvesicles that had elevated cancer-associated fibroblast markers in 16.66% of the studies. In addition, TMVs in treated HGFs produced more glucose and lactate while expressing greater levels of GLUT1, PDK1, and MCT4 and lower levels of Caveolin 1. When CAL-27 xenograft along with untreated and TMV-treated HGFs was injected into mice, TMV treatment resulted in a significant increase in tumor weight. OSCC cells, when cultured with TMV-treated HGFs, increased their invasive ability, which was partially reversed by knocking down monocarboxylate transporter 1 in CaL-27 cells [35,36]. Rabinowits et al. found a significant difference in nine miRNAs that were differently upregulated in malignant tissue, and seven were downregulated in benign tissue. The upregulation of miRNA 130-3p might be due to the overexpressed miRNA130-3p in OSCC, which binds to three untranslated region sequences of peroxisome proliferator-activated receptors and PTEN deleted on chromosome 10, represses their expression, and shows upregulated miRNA-21 [32]. The nuclear factor kappa B (NF-κB) pathway may be activated by extracellular vesicle-associated miRNA-21, which would then induce inflammation in monocytes. miRNA-21 involved in antiapoptotic activity is expressed in tumors, and higher expression indicates tumor growth. NF-κB activation is part of the immune system that targets and removes altered cells. However, the roles of other miRNAs are still unexplored. Extracellular vesicles from cancer cells can transfer miRNA-21 and may promote migration and invasion by modulating the tumor microenvironment. CD63 plasma extracellular vesicle levels were not substantial, most likely because of postsurgery inflammatory reactions. Additionally, certain patterns indicated a link between poor survival and persistently high plasmatic CD63 extracellular vesicle levels after resection [37,38]. CD63 markers on various extracellular vesicles can detect the load in saliva or blood and assist in early detection. Liquid biopsies can be easily isolated from bodily fluids like saliva, blood, and so forth. Exosomes are richer in concentrations than circulating tumor cells, and circulating tumor DNA has more than 109 vesicles/mL. It has inherent stability and maintains the integrity of its contents. Exosomes are studied both in saliva and circulation (plasma and serum). There is not much scientific evidence to prove the best source of exosomes; however, it can be used to predict the progression and outcome of treatment.

Isolation of exosomes has different methods based on density ultracentrifugation and density gradient centrifugation, based on size (ultrafiltration and size exclusion chromatography), and based on function (immunoaffinity capture chromatography, polymer-based precipitation, microfluidics, chip-based technologies were found) [39]. Ultracentrifugation and polymer-based precipitation are most commonly used in the studies included in this review [40,41].

Characterization of exosomes is necessary before analysis, based on molecular features characterized by western blot, flow cytometry, and immunosorbent assay. Electron microscope imaging, atomic force microscopy, nano tracking analysis, photon correlation spectroscopy, and adjustable resistive pulse sensing are used for characterizing the physical features of exosomes [42]. Exosomes are based on both molecular and physical characteristics in all the included studies, and western blotting, nano-tracking analysis, and electron imaging are most commonly used in the studies included in this review. The majority of the studies analyzed the molecular expression using quantitative polymerase chain reaction, followed by microarray, and the remaining studies were analyzed using the immunocapture method. Based on the analysis of five articles included in the review, three studies were related to plasma exosomes, and the remaining studies were related to serum exosomes. When patients with OSCC were compared to healthy control patients, there was a significant difference in the expression of the molecular markers included in the research. Furthermore, miRNA, GLUT1, PDK1, caveolin 1, and cric_00001999 showed a correlation with OSCC. Hence, miRNA plays an important role in OSCC diagnosis. Earlier identification of these can offer a good prognosis for the patients.

Limitations

The limitations of the included studies are low sample size, heterogeneity, different methods of isolation, and characterization of exosomes. The limitations of this review include only English literature, minimal literature evidence on circulating exosomes in OSCC (there are few original papers reviewed), and different approaches and markers employed in the detection of OSCC.

Implications in Practice

Considering the significant results, molecular analysis of circulating exosomes with further validated studies could make circulating exosomes a part of the diagnostic protocol for OSCC for early patient diagnosis.

Implications for Future Research

Further studies are warranted to evaluate the molecular markers in circulating exosomes derived from patients with OSCC to gain further insights into their role in oral carcinogenesis. Studies with large sample sizes and OPMDs like oral submucous fibrosis and leukoplakia will be helpful in understanding the transitions of OPMDs to OSCC.

## Conclusions

Circulating exosome expression of miRNA27, miRNA21, and miR155 showed significant upregulation in patients with OSCC. Understanding the role of molecular markers from circulating exosomes in pathogenesis will provide a better understanding of lesion development, but more study is needed in this area. Further studies are needed to conclude whether circulating exosomes can be used for early diagnosis of OSCC.

## Appendices

**Table 4:** PRISMA checklist PRISMA: Preferred Reporting Items for Systematic Reviews and Meta-Analyses

Section and topic	Item #	Checklist item	The location where the item is reported
Title			
Title	1	Identify the report as a systematic review.	1
Abstract			
Abstract	2	See the PRISMA 2020 for the Abstracts checklist.	1
Introduction			
Rationale	3	Describe the rationale for the review in the context of existing knowledge.	2-3
Objectives	4	Provide an explicit statement of the objective(s) or question(s) the review addresses.	2-3
Methods			
Eligibility criteria	5	Specify the inclusion and exclusion criteria for the review and how studies were grouped for the syntheses.	3-4
Information sources	6	Specify all databases, registers, websites, organizations, reference lists, and other sources searched or consulted to identify studies. Specify the date when each source was last searched or consulted.	3
Search strategy	7	Present the full search strategies for all databases, registers, and websites, including any filters and limits used.	3
Selection process	8	Specify the methods used to decide whether a study met the inclusion criteria of the review, including how many reviewers screened each record and each report retrieved, whether they worked independently, and if applicable, details of automation tools used in the process.	4
Data collection process	9	Specify the methods used to collect data from reports, including how many reviewers collected data from each report, whether they worked independently, any processes for obtaining or confirming data from study investigators, and if applicable, details of automation tools used in the process.	4
Data items	10a	List and define all outcomes for which data were sought. Specify whether all results that were compatible with each outcome domain in each study were sought (e.g. for all measures, time points, analyses), and if not, the methods used to decide which results to collect.	4
	10b	List and define all other variables for which data were sought (e.g. participant and intervention characteristics, funding sources). Describe any assumptions made about any missing or unclear information.	4
Study risk of bias assessment	11	Specify the methods used to assess the risk of bias in the included studies, including details of the tool(s) used, how many reviewers assessed each study and whether they worked independently, and if applicable, details of automation tools used in the process.	4
Effect measures	12	Specify for each outcome the effect measure(s) (e.g. risk ratio, mean difference) used in the synthesis or presentation of results.	4
Synthesis methods	13a	Describe the processes used to decide which studies were eligible for each synthesis (e.g. tabulating the study intervention characteristics and comparing against the planned groups for each synthesis (item #5)).	4
	13b	Describe any methods required to prepare the data for presentation or synthesis, such as handling of missing summary statistics, or data conversions.	4
	13c	Describe any methods used to tabulate or visually display the results of individual studies and syntheses.	4
	13d	Describe any methods used to synthesize results and provide a rationale for the choice(s). If meta-analysis was performed, describe the model(s), method(s) to identify the presence and extent of statistical heterogeneity, and software package(s) used.	Na
	13e	Describe any methods used to explore possible causes of heterogeneity among study results (e.g., subgroup analysis, meta-regression).	Na
	13f	Describe any sensitivity analyses conducted to assess the robustness of the synthesized results.	Na
Reporting bias assessment	14	Describe any methods used to assess the risk of bias due to missing results in a synthesis (arising from reporting biases).	Na
Certainty assessment	15	Describe any methods used to assess certainty (or confidence) in the body of evidence for an outcome.	4-5
Results			
Study selection	16a	Describe the results of the search and selection process, from the number of records identified in the search to the number of studies included in the review, ideally using a flow diagram.	5
	16b	Cite studies that might appear to meet the inclusion criteria, but which were excluded, and explain why they were excluded.	5
Study characteristics	17	Cite each included study and present its characteristics.	5
Risk of bias in studies	18	Present assessments of risk of bias for each included study.	6
Results of individual studies	19	For all outcomes, present, for each study: (a) summary statistics for each group (where appropriate) and (b) an effect estimate and its precision (e.g., confidence/credible interval), ideally using structured tables or plots.	6
Results of syntheses	20a	For each synthesis, briefly summarise the characteristics and risk of bias among contributing studies.	7
	20b	Present results of all statistical syntheses conducted. If meta-analysis was done, present for each the summary estimate and its precision (e.g., confidence/credible interval) and measures of statistical heterogeneity. If comparing groups, describe the direction of the effect.	Na
	20c	Present results of all investigations of possible causes of heterogeneity among study results.	Na
	20d	Present results of all sensitivity analyses conducted to assess the robustness of the synthesized results.	Na
Reporting biases	21	Present assessments of risk of bias due to missing results (arising from reporting biases) for each synthesis assessed.	Na
Certainty of evidence	22	Present assessments of certainty (or confidence) in the body of evidence for each outcome assessed.	8
Discussion			
Discussion	23a	Provide a general interpretation of the results in the context of other evidence.	9-12
	23b	Discuss any limitations of the evidence included in the review.	9-12
	23c	Discuss any limitations of the review processes used.	9-12
	23d	Discuss the implications of the results for practice, policy, and future research.	9-12
Other information			
Registration and protocol	24a	Provide registration information for the review, including the register name and registration number, or state that the review was not registered.	3
	24b	Indicate where the review protocol can be accessed, or state that a protocol was not prepared.	Na
	24c	Describe and explain any amendments to the information provided at registration or in the protocol.	3
Support	25	Describe sources of financial or non-financial support for the review, and the role of the funders or sponsors in the review.	Provided
Competing interests	26	Declare any competing interests of review authors.	Provided
Availability of data, code, and other materials	27	Report which of the following are publicly available and where they can be found: template data collection forms; data extracted from included studies; data used for all analyses; analytic code; any other materials used in the review.	Provided
